# Evidence of Henipavirus Infection in West African Fruit Bats

**DOI:** 10.1371/journal.pone.0002739

**Published:** 2008-07-23

**Authors:** David T. S. Hayman, Richard Suu-Ire, Andrew C. Breed, Jennifer A. McEachern, Linfa Wang, James L. N. Wood, Andrew A. Cunningham

**Affiliations:** 1 Institute of Zoology, Zoological Society of London, London, United Kingdom; 2 Cambridge Infectious Diseases Consortium, University of Cambridge, Cambridge, United Kingdom; 3 Wildlife Division of the Forestry Commission, Accra, Ghana; 4 School of Veterinary Science, The University of Queensland, Brisbane, Queensland, Australia; 5 Australian Biosecurity Cooperative Research Centre, Geelong, Victoria, Australia; 6 CSIRO Livestock Industries, Australian Animal Health Laboratory, Geelong, Victoria, Australia; U.S. Naval Medical Research Center Detachment/Centers for Disease Control, United States of America

## Abstract

Henipaviruses are emerging RNA viruses of fruit bat origin that can cause fatal encephalitis in man. Ghanaian fruit bats (megachiroptera) were tested for antibodies to henipaviruses. Using a Luminex multiplexed microsphere assay, antibodies were detected in sera of *Eidolon helvum* to both Nipah (39%, 95% confidence interval: 27–51%) and Hendra (22%, 95% CI: 11–33%) viruses. Virus neutralization tests further confirmed seropositivity for 30% (7/23) of Luminex positive serum samples. Our results indicate that henipavirus is present within West Africa.

## Introduction

Henipaviruses are emerging fatal zoonotic RNA viruses with *Pteropus* spp. fruit bats identified as their reservoir hosts [1]. To date, viruses in this genus have been isolated from bats only in Australia (Hendra virus - HeV) [2] and Asia (Nipah virus - NiV) [3], although there is recent serological evidence of infection in bats in Madagascar [4]. There has been transmission to humans through horse [5] and pig [6] intermediate hosts and direct bat to human transmission, followed by human-to-human transmission [7], [8] . Henipaviruses are important emerging pathogens of humans; for example, in Malaysia in 1999, over one million pigs were culled to control an outbreak which killed 105 people [9].

The distribution of *Pteropus* spp. bats was assumed to limit henipavirus distribution. In Madagascar, however, henipavirus antibodies were found in the non-pteropid bats, *Eidolon dupreanum*, and *Rousettus madagascariensis*, but both species were sympatric with seropositive pteropid bats [4]. Here we report the results of serological surveys for henipavirus infection in fruit bats in Ghana, a country in West Africa approximately 5800 km from the nearest pteropid bat populations.

## Results

The numbers of each species of bat tested and the serology results using a Luminex binding assay [10] are presented in Table 1. Evidence of infection with henipavirus was common in *E. helvum*, with 23 of 59 (39%, 95% CI: 27–51%) showing reactivity to henipavirus: 23 showed reactivity to NiV (39%, 95% CI: 27–51%) and 13 showed reactivity to HeV (22%, 95% CI: 11–33%). All the HeV-seropositive bats showed reactivity to both viruses. Of the 23 *E. helvum* samples seropositive using the Luminex binding assay, seven were positive using virus neutralization tests (VNTs) (3 for NiV only, 1 for HeV only, 3 for both viruses).

**Table 1:** Details of the bat species and their respective seroprevalence rates calculated using the Luminex binding assay data.

Species	Habitat where caught	Number tested	Number positive (seroprevalence (%), 95% CI)		Percentage adult
			Hendra	Nipah	
*Epomophorus gambianus*	Open woodland*	89	0	1 (1, 0–3)	62
*Eidolon helvum*	City colony**	59	13 (22, 11–33)	23 (39, 27–51)	95
*Epomops franqueti*	Forest**	29	0	0	77
*Epomops buettikoferi*	Forest**	7	0	0	85
*Hypsignathus monstrosus*	Forest**	18	0	1 (6, 0–16)	56
*Nanonycteris veldkampii*	Forest**	4	0	0	100

* *E. gambianus* was caught in all habitats, including at the city colony and in plantation.

** A small number was caught in plantation.

One serum sample from each of *E. gambianus* (1%, 0–3% CI) and *H. monstrosus* (6%, 0–16%) gave positive readings for NiV using the Luminex binding assay, but gave negative results with the VNT. No other bats gave seropositive results for henipavirus infection.

**Figure 1:**
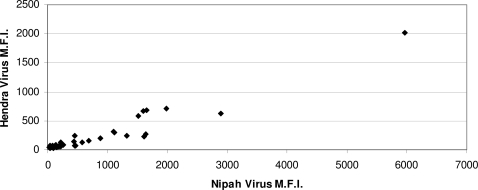
Serological cross-reactivity in *E. helvum* between HeV and NiV. The Luminex assay MFI readings against each of the NiV and HeV G proteins were plotted along the X and Y axis, respectively. Note: the scale is different for the two axes.

Many of the *E. helvum* tested were positive to both HeV and NiV (Table 1). The degree of cross-reactivity against both henipaviruses within individual positive sera is illustrated in Figure 1. For NiV, there was no significant association between gender and seropositivity in the *E. helvum* sampled (9 of 19 females were seropositive compared with 14 of 40 males; χ^2^ = 0.4, p = 0.5), but for HeV, females were marginally significantly more likely to be seropositive (7 of 19 females were seropositive compared with 6 of 40 males; Fishers exact test p = 0.09).

As a high level of seropositivity was detected in *E. helvum*, we attempted to determine a possible case reproduction rate (R_0_) for henipavirus infection in this species using:
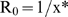
where x* = proportion of susceptible hosts in a population [11].

This attempt assumes that infection with henipavirus within the bat population is endemic, stable, and randomly dispersed, that lifelong immunity is detectable serologically and all seropositive animals have lifelong immunity and that seropositivity is to a single virus (Luminex binding assay results for NiV were used). Based on these assumptions, R_0_ = 1.6 (95% CI: 1.3–2.0).

## Discussion

We found seropositivity to henipavirus in *E. helvum* fruit bats in Ghana, providing the first evidence of henipavirus infection in Africa. Mainland Africa has no *Pteropus* spp. fruit bats [12], thus this is the first demonstration of henipavirus antibodies in animals not sympatric with pteropid bats. *E. helvum* is widely distributed across sub-Saharan Africa. It is known to inhabit the equatorial tropical forest region and is believed to migrate into savannah regions annually [12], [13]. The high seroprevalence and distance from any *Pteropus* species would indicate that a member of the genus *Henipavirus* has spread through, or is circulating in, the *E. helvum* population in Accra, the capital city of Ghana. The identity of the infecting virus (or viruses) that elicited the antibody response to henipavirus remains unknown.

As in studies of henipaviruses in pteropid bats [10], [14], cross reactivity to HeV and NiV was found (Figure 1). The cross reaction of the positive sera to HeV and NiV may be a feature of the virus or of the reactivity of *E. helvum* antibodies. It is worth noting that most of the serum samples gave a higher reading for NiV than HeV, indicating the virus(es) circulating in the West African *E. helvum* populations is more NiV-like. The seropositive animals were apparently healthy. This suggests that *E. helvum* might be similar to pteropid bats, surviving infection and possibly acting as a reservoir host.

Two bats from other species were seropositive for NiV using the Luminex binding assay, but were negative using the VNT. The sample from *H. monstrosus* had an MFI just above the cut-off, and not enough individuals were caught to evaluate seroprevalence. *Epomophorus gambianus*, however, was caught in large numbers and the seropositive individual exhibited a high binding MFI to NiV.

Although the Luminex binding assay used in this study has not been stringently validated with bat sera due to lack of the required number of known positive and negative bat sera from different species, it has been extensively tested with known positive and negative sera of other species and shown to perform better than conventional ELISA-based binding assays in terms of both sensitivity and specificity [10]. For *E. helvum* sera, henipavirus seropositivity was confirmed in around one third of the Luminex positive samples using VNT. The lower seroprevalence detected using VNT is most likely due to a low level of antibodies circulating in the bat population (as indicated by the low VNT titres, varying from 1∶10 to 1∶80). The fluorescence-based Luminex assay is much more sensitive than the conventional VNT, and hence is expected to pick up more positive samples. Another possibility is the presence of more than one henipavirus species in *E. helvum*, antibodies to one of which are unable to neutralise either HeV or NiV, but can cross react with their G proteins in the Luminex assay. This is less likely due to the fact that the G proteins of henipaviruses are responsible for receptor binding and, as in most other paramyxoviruses, the target of neutralizing antibodies. It is therefore expected that G-reactive antisera would neutralize live virus if their G-reacting antibodies are at a sufficiently high level.

The difference in henipavirus seroprevalence between *E. gambianus* and *E. helvum* raises the question as to whether this is a feature of species susceptibility or of species ecology. *E. helvum* lives in large, densely populated colonies numbering hundreds of thousands: the urban colony sampled in this study comprised at least 500,000 bats. This is in comparison to the much smaller (tens or hundreds) and less dense colonies of *E. gambianus*, thus populations of this species would be less likely to sustain henipavirus infection. There is, however, genetic evidence that *Eidolon* spp. differ from other African fruit bats [15]. African fruit bats form an endemic clade within fruit bat phylogeny, with the exception of *Eidolon*. The *Eidolon* genus has been found to be more closely related to other genera, including *Pteropus*, than to the other proposed African clades; African *Rousettus*, Epomophorine and Myonycterine [15].

Although caution is needed in interpretation, the R_0_ value for henipavirus in the *E. helvum* colony sampled in this study was higher than those estimable from other seroprevalence datasets for henipavirus in pteropid bats [4], [14]. A higher value might reflect innate host-species or virus differences, or simply reflect the high contact rate in this highly gregarious species.

Further to the serological results obtained in this study, work is now underway to confirm the presence of henipavirus infection in African bats using reverse transcriptase PCR and virus isolation. This is also necessary to characterize the virus(es) concerned and to make comparisons with henipaviruses found in Australian and Asian *Pteropus* spp. fruit bats. An additional priority for future research is the strengthening of medical surveillance for encephalitis in Africa and the investigation of henipavirus involvement in patients suffering from encephalitis, particularly where alternative diagnoses, such as rabies and cerebral malaria, have not been confirmed.

In conclusion, serological evidence for henipavirus infection in *E. helvum* in Ghana poses interesting questions regarding henipavirus ecology within African bat populations and its potential for zoonotic emergence. *E. helvum* is widely distributed across sub-Saharan Africa where it commonly forms extremely large colonies in close proximity to both man and domestic animals. *E. helvum* is also a common and important source of bushmeat in West Africa, thus presenting another possible conduit for zoonotic emergence.

## Methods

Bats were sampled during two visits in January and May 2007 at six sites across Ghana: the centre of Accra (urban habitat – the capital city of Ghana), woodland on the outskirts of Accra (savannah habitat), and in forest habitat at Pra, Kibi, Adoagyiri and Oyibi. The last sampling site was in a plantation along a woodland/forest border. All sites were within 180 km of each other. Bats were captured either using 6–18 m mist nets or, for roosting *E. helvum*, using nets on poles. Up to 1% of body weight of blood was taken from the propatagial vein prior to release. A total of 206 bats of six species were caught, sampled and tested (Table 1).

Two species, *Epomophorus gambianus* (n = 89) and *E. helvum* (n = 59), were tested in sufficient numbers for reasonable inferences to be made about seroprevalence rates: 59 being the sample size required to have 95% confidence of finding at least one seropositive in a large population given a 5% seroprevalence, assuming random sampling [16]. Ninety five per cent confidence intervals for seroprevalences were calculated using a standard approach [16]. All but three *E. helvum* samples were derived from the colony in central Accra, whereas *E. gambianus* was sampled across all habitats. We assumed the sampled *E. gambianus* and *E. helvum* were from single metapopulations.

Sera were tested for antibodies binding to the recombinant HeV and NiV G proteins in a Luminex multiplexed binding assay [10]. The recombinant G proteins used in the Luminex assay were generated using a mammalian expression system in a soluble form by removing the transmembrane domain [17]. The soluble G proteins retained their ability to bind the cellular receptor molecule, indicating their native conformation was maintained, which is important for the detection of neutralizing antibodies. Samples showing positive binding in the Luminex assay were further confirmed by a virus neutralization test (VNT) for both HeV and NiV [10]. For the Luminex binding assay, bat sera with median fluorescence intensities (MFI) readings of ≥200 were considered positive. Three times the average background reading of negative sera was used as a cut-off for the binding assay. For VNT, sera with a VNT titre of ≥1∶10 were considered positive.
